# Optimal volume of injectate for fluoroscopy-guided cervical interlaminar epidural injection in patients with neck and upper extremity pain

**DOI:** 10.1097/MD.0000000000005206

**Published:** 2016-10-28

**Authors:** Jun Young Park, Doo Hwan Kim, Kunhee Lee, Seong-Soo Choi, Jeong-Gil Leem

**Affiliations:** Department of Anesthesiology and Pain Medicine, Asan Medical Center, University of Ulsan College of Medicine, Seoul, Republic of Korea.

**Keywords:** cervical interlaminar epidural injections, contrast dye spread, neck and upper extremity pain

## Abstract

There is no study of optimal volume of contrast medium to use in cervical interlaminar epidural injections (CIEIs) for appropriate spread to target lesions. To determine optimal volume of contrast medium to use in CIEIs. We analyzed the records of 80 patients who had undergone CIEIs. Patients were divided into 3 groups according to the amount of contrast: 3, 4.5, and 6 mL. The spread of medium to the target level was analyzed. Numerical rating scale data were also analyzed. The dye had spread to a point above the target level in 15 (78.9%), 22 (84.6%), and 32 (91.4%) patients in groups 1 to 3, respectively. The dye reached both sides in 14 (73.7%), 18 (69.2%), and 23 (65.7%) patients, and reached the ventral epidural space in 15 (78.9%), 22 (84.6%), and 30 (85.7%) patients, respectively. There were no significant differences of contrast spread among the groups. There were no significant differences in the numerical rating scale scores among the groups during the 3 months. When performing CIEIs, 3 mL medication is sufficient volume for the treatment of neck and upper-extremity pain induced by lower cervical degenerative disease.

## 1 Introduction

Chronic neck and upper-extremity pain are common in the general adult population^[^1–3^]^ with a lifetime prevalence of 26% to 71%.^[^4 5^]^ Chronic neck and upper-extremity pain may be related to changes in intervertebral discs, spinal nerves, cervical facet joints, ligaments, fascia, and muscles.^[^1–3^]^ The most common causes of neck and upper-extremity pain associated with spinal nerve lesions are cervical spinal stenosis and herniated intervertebral discs (HIVDs).^[^1–4 6^]^

Various therapies are used to treat such pain, including physical therapy, analgesia, and epidural injections of steroid.^[^2–5 7^]^ Despite the ongoing debate regarding long-term efficacy, cervical interlaminar epidural injections (CIEIs) of steroids have been shown to be effective for treating chronic neck and upper-extremity pain, as demonstrated by randomized clinical trials and systematic reviews.^[^3 8^]^ Cervical transforaminal epidural injections (CTFEIs) may be more effective than CIEIs. However, the difference in effect between CTFEIs and CIEIs is small, and there have been no prospective studies on this. Moreover, the neurological complications of CTFEIs are more catastrophic than those of CIEIs. Therefore, CIEIs are a first-line intervention for chronic pain of the neck and upper extremities.^[^8–10^]^

The effects of CIEIs are determined by the spread of medications in the epidural space. Generally, this spread is affected by various factors, such as the administered volume, insertion site, the anatomical variance of the epidural space, the site of adhesion or compression, pregnancy, age, height, and weight.^[11]^ Among such factors, the volume administered and injection site are known to be a major factors determining the efficacy of the injected medication.^[^11 12^]^

In clinical practice, 2 to 7 mL medication administered via CIEI is thought to be an adequate volume to spread throughout the epidural space in degenerative cervical spinal disease.^[^2 13–15^]^ However, there is no consensus on the optimal volume of medication for cervical epidural steroid injections. There have been few studies on the relationship between the volume of solution and the cephalad spread of solution in CIEIs.^[^14 16^]^ Moreover, most lesion sites associated with neck and upper-extremity pain are in the ventral epidural space.^[3]^ Therefore, it is important to identify the optimal volume of medication to use for appropriate spread to ventral epidural space at the lesion level when performing CIEIs.^[3]^ However, few studies have addressed this.^[15]^

In our present study, we evaluated epidurography contrast patterns in fluoroscopically guided CIEIs and determined the optimal volume for cephalad spread, bilaterally spread, and spread in the ventral epidural space at the lesion level.

## 2 Methods

### 2.1 Patients

This single-center, retrospective observational study used the institutional registry records of 80 patients who underwent CIEIs of steroids between January and August of 2015. The Local Ethics Board of Asan Medical Center approved this study (approval number, 2015–122).

Adult patients with neck or upper-extremity pain and confirmed degenerative cervical disease at the C5 to C7 levels who had also undergone magnetic resonance imaging (MRI) before therapeutic CIEIs of steroids were included in the study. Patients were excluded from the study if there was severe cervical spinal stenosis indicated by MRI, prior cervical spine surgery, or uncontrolled medical disease. Patients with contraindications for CIEIs, such as coagulopathy, infection, or allergy to lidocaine, contrast, or steroids were also excluded. All patients were divided into 3 groups according to the volume of diluted contrast dye administered (3, 4.5, and 6 ml). Group 1 received 3 mL (2 mL normal saline and 1 mL IOHEXOL 300 mg/mL [OMNIPAQUE^TM^ GE Healthcare, Shanghai, China]). Group 2 received 4.5 mL (3 mL normal saline and 1.5 mL IOHEXOL 300 mg/mL). Group 3 received 6 mL (4 mL normal saline and 2 mL IOHEXOL 300 mg/mL).

### 2.2 Interventions

**Figure 1:**
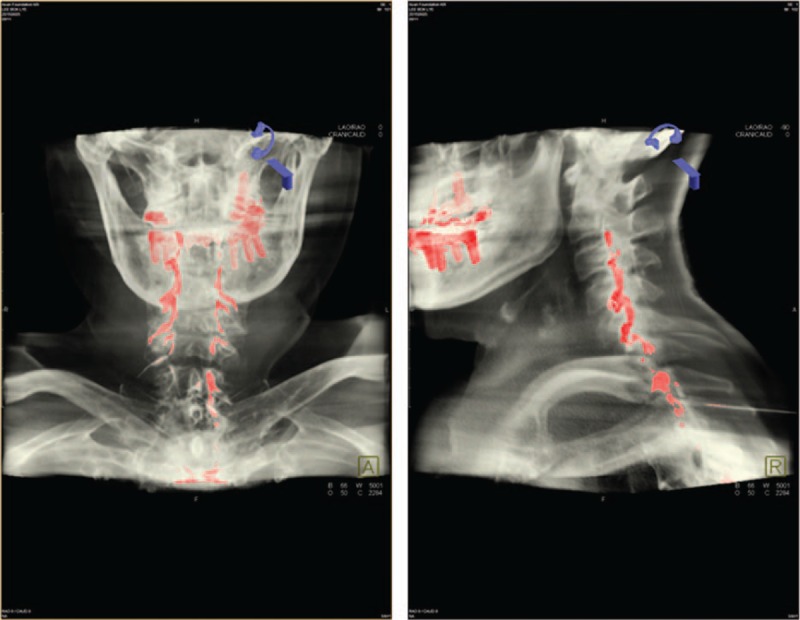
Fluoroscopic 3D reconstructed image of an epidurogram of 6.0 mL contrast dye. (A) Contrast dye spread at the C3 upper endplate in a 3D reconstructed anteroposterior view image. (B) Contrast dye spread into the ventral epidural space at the C3 upper endplate in a 3D reconstructed lateral view image. 3-dimensional = 3D.

**Figure 2:**
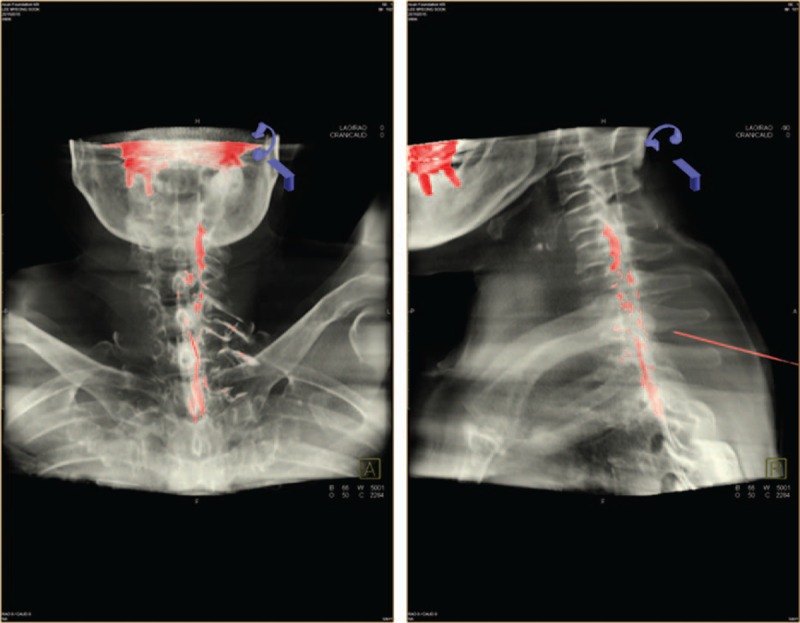
Fluoroscopic 3D reconstructed image of an epidurogram of 3.0 mL contrast dye. (A) Contrast dye spread at the C5 upper endplate in a 3D reconstructed anteroposterior view image. (B) Contrast dye spread into the ventral epidural space at the C5 upper endplate in a 3D reconstructed lateral view image. 3-dimensional = 3D.

CIEIs were performed under fluoroscopic guidance with equipment to monitor blood pressure, pulse rate, and pulse oximetry. Patients were placed in a prone position with a pillow under the chest, and the neck was flexed. Then the head was rested on a table and the arms were positioned at the side. Using an anteroposterior (AP) view, the C7 to T1 vertebrae were identified. After sterile preparation and draping of the insertion area, the skin was infiltrated with 1% lidocaine, and a 21G Tuhoy needle was gently advanced at the paramedian site of the lower T1 and advanced to the C7 to T1 interspinous space under fluoroscopic guidance. The needle was advanced to a place posterior to the ventral interlaminar line under the contralateral oblique (CLO) view.^[^17 18^]^ Then the needle was cautiously advanced, using the loss of resistance method, with an air-filled syringe. Subsequently, 0.2 mL contrast dye was injected to confirm the epidural space. A diluted contrast dye was then injected via an epidural needle under real-time fluoroscopic guidance, and the extent of spread was determined by fluoroscopic AP and CLO images and fluoroscopic 3-dimensional reconstructed images of the cervical spine (Figs. 1 and 2).

The following characteristics were recorded: contrast spread to below the target level or above the target level, spread to the ipsilateral side or to both sides at the target level, and spread confined to the dorsal epidural space or the ventral epidural space at the target level. All CIEIs were performed at the C7 to T1 segments. All target level was determined by MRI before CIEI. Therefore, all CIEIs were performed at a level 1 or 2 vertebra below the target lesion, and cephalad spread was determined based on whether the contrast dye reached a point above than the lesion. Spread to the ipsilateral side or both sides of contrast dye spread were determined based on whether or not the dye reached both intervertebral foramina at the target level. Spread to the ventral epidural space was determined based on whether the dye was confined to the dorsal epidural space or spread beyond that to the ventral epidural space at the target level.

After a cervical epidurogram was obtained, 1% lidocaine with 5 mg dexamethasone was injected with an equivalent volume of contrast dye. Numerical rating scale (NRS) data were collected at baseline and at 1 and 3 months follow-up.

### 2.3 Statistical analysis

Statistical analyses were performed using the statistical package SPSS 21.0 for Windows (SPSS Inc., Chicago, IL). Demographic data were compared using chi-squared or 1-way analysis of variance, as appropriate. Data are reported as mean ± SD or number of patients. The spread of contrast dye was analyzed using a chi-square test or Fisher exact test, as appropriate. A *P* value <0.05 was considered significant. As data loss resulting from missing values in electronic databases was expected, the linear mixed effect model was used to compare changes within and between groups in terms of NRS pain scores at baseline and at 1 and 3 months post-CIEI.

## 3 Results

In total, 80 patient records were analyzed and categorized into group 1 (19 patients), group 2 (26 patients), and group 3 (35 patients). The demographic characteristics of the patients are presented in Table 1. There were no significant differences in age, sex, height, weight, diagnosis, target level of treatment, or needle approach between the groups.

**Table 1:**
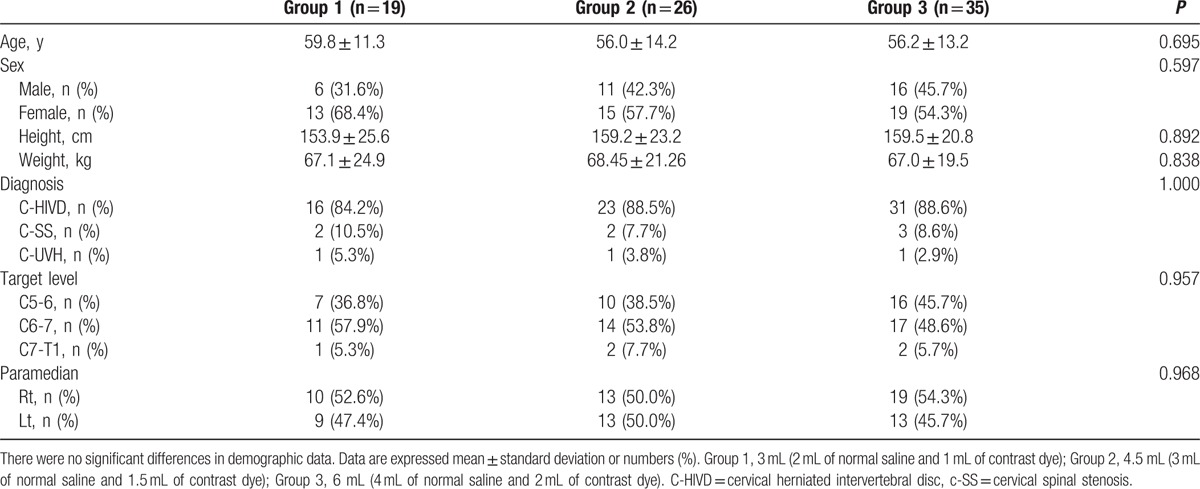
Demographics data and clinical characteristics of the population (n = 80).

Based on MRI, the target level was determined before CIEI. During CIEI, the contrast dye had spread to a point above the target level were 15 (78.9%), 22 (84.6%), and 32 (91.4%) patients in groups 1, 2, and 3, respectively (Table 2). There were no significant differences among the groups (*P* = 0.385). The dye reached both sides at target level were in 14 (73.7%), 18 (69.2%), and 23 (65.7%) patients, respectively (Table 3), with no significant differences among groups (*P* = 0.832), and reached the ventral epidural space at target level were in 15 (78.9%), 22 (84.6%), and 30 (85.7%) patients, respectively (Table 4), with no significant differences among the groups (*P* = 0.860).

**Table 2:**
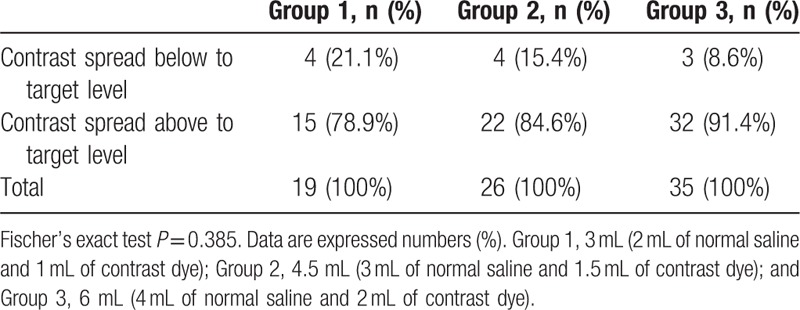
Contrast spread cephalad to target level in the cervical interlaminar injection.

**Table 3:**
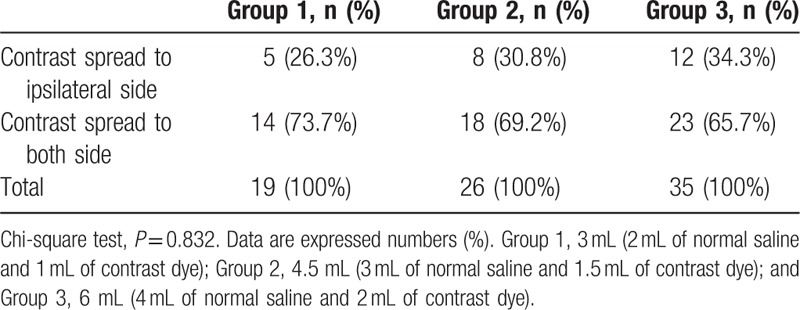
Contrast spread to both side in the cervical interlaminar injection.

**Table 4:**
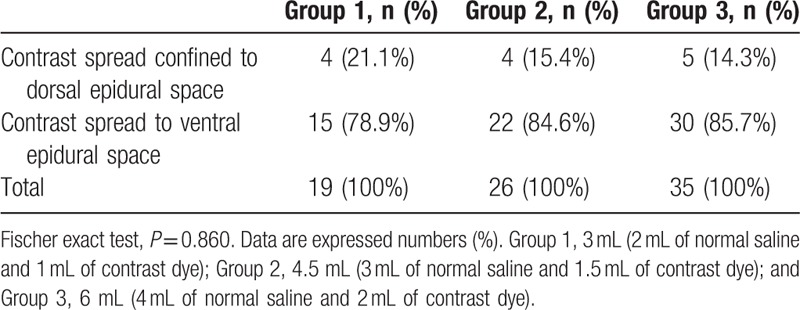
Contrast spread to ventral epidural space in the cervical interlaminar injection.

NRS values at baseline and 1 and 3 months after CIEI are listed in Table 5. In all groups, significant differences in NRS score were observed at 1 month after CIEI compared to baseline (*P* *<* 0.002 in group 1, *P* < 0.001 in group 2, and *P* < 0.001 in group 3). There were no significant differences at 3 months compared to 1 month in any group *(P* *=* 0.682, *P* = 0.929, and *P* = 1.000, respectively). Linear mixed effect model analysis indicated that the adjusted prediction of the NRS score at baseline was 6.89 [95% confidence interval (CI): 6.05–7.74] for group 1, 6.58 (95% CI: 5.82–7.34) for group 2, and 6.69 (6.06–7.31) for group 3. At 1 month after CIEI, the adjusted NRS score was 4.80 (95% CI: 3.63–5.97) for group 1, 4.29 (95% CI: 3.35–5.22) for group 2, and 4.30 (95% CI: 3.51–5.08) for group 3. At 3 months, it was 4.92 (95% CI: 3.40–6.44) for group 1, 4.47 (95% CI: 3.10–5.84) for group 2, and 4.42 (95% CI: 2.97–5.87) for group 3. There were no significant differences between the groups at any time point.

**Table 5:**
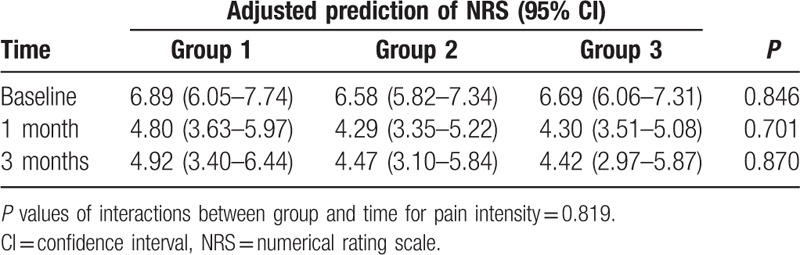
Adjusted predictions of pain intensity scores after cervical epidural steroid injections from baseline for each group.

## 4 Discussion

We investigated the spread patterns of contrast dye after CIEI performed at segments C7 to T1 according to injection volume in patients with chronic neck and upper-extremity pain. The patterns did not significantly differ by injected volume. Chronic neck and upper-extremity pain are common.^[^1–3^]^ Similar to acute and chronic low back pain, neck pain also results in considerable socioeconomic and healthcare costs.^[19]^ Furthermore, a substantial number of patients experience severe chronic neck pain, and the lifetime prevalence of neck and upper-extremity pain has been reported to be 26% to 71%.^[^4 5^]^ Chronic neck and upper-extremity pain are associated with various structures such as intervertebral discs, spinal nerves, cervical facet joints, ligaments, fascia, and muscles.^[^1–3^]^ The most common contributors to such pain associated with spinal nerve lesions are cervical spinal stenosis and HIVDs.^[^1–4 6^]^

Various therapies are used to treat such pain.^[^3 4^]^ Initial treatment typically consists of conservative strategies such as rest, physiotherapy, and medications.^[^2–5^]^ However, cervical steroid injections are frequently used for patients whose pain is refractory to conservative management.^[^20 21^]^ Despite debate over long-term efficacy, CIEIs of steroids are known to effectively treat conditions of neck and upper-extremity pain in cervical degenerative diseases, as demonstrated by randomized clinical trials and systematic reviews.^[^3 8^]^ Particularly when cervical disc herniation or cervical spinal stenosis are associated with neck and upper-extremity pain, CIEIs of steroids are widely used.^[^8 20 22 23^]^

When CIEIs are performed, the C6 to 7 or C7 to T1 sites are preferred, because degenerative cervical diseases occur most commonly at vertebral segments below C5, such as C5 to 6 and C6 to 7.^[^24–26^]^ Moreover, CIEIs are associated with a rare risk of catastrophic neurological injury. C6 to 7 and C7 to T1 are thought to have a greater epidural space than the rest of the cervical spine.^[^27 28^]^ Therefore, it is recommended to perform CIEIs at C7 to T1, but preferably not higher than C6 to 7.^[26]^ In clinical practice, the sites C6 to 7 and C7 to T1 are more effective and safer than other levels.^[^27 28^]^ In this study, we performed CIEIs only at C7 to T1 because the spread of contrast dye may be affected by injected site.

For CIEIs to be effective, medication must spread to the site of nerve pathology.^[1]^ This spread is affected by various factors, such as the volume of medication administered, the site of needle insertion, anatomical variance of the epidural space, epidural adhesion or compression, pregnancy, age, height, and weight.^[11]^ The administered volume and injection site are major factors determining the efficacy of injected medication.^[^11 12^]^

To treat pain, it is important for medication to spread to a higher position than a suspected lesion. In clinical practice, 2 to 7 mL medication during performing CIEIs is typically considered an adequate volume for the treatment of pain induced by degenerative cervical spinal disease.^[^2 13^]^ However, there is no consensus on the optimal volume of medication for such injections. Larger volumes have potential risks such as increased CSF pressure, headache, and local-anesthetic toxicity.^[^13 29 30^]^ Moreover, the dilution of steroids caused by larger volumes of local anesthetics may be insufficient to treat pain.^[^5 11^]^

There have been some studies on contrast pattern and volume in CIEIs.^[^12 15 16^]^ Yokoyama et al^[12]^ showed that the contrast pattern is useful for predicting the distribution of local anesthetics. Other studies have shown that a 2 to 4 mL volume is adequate for contrast spread throughout the entire cervical epidural space, bilaterally.^[^15 16^]^ In 1 study, a 5 mL volume was found to be optimal for distribution to the lower cervical spine in degenerative cervical spinal diseases.^[14]^ However, these studies had some limitations. In some study, cervical lesions such as adhesions, HIVDs, and spinal stenosis were not described, although such lesions may affect the spread of contrast dye.^[15]^ In another study, the injection site, which can also affect spread, was not uniform.^[16]^ Moreover, most lesion sites associated with neck and upper-extremity pain are in the ventral epidural space, such as the spinal nerve, and intervertebral disc.^[3]^ Therefore, when performing CIEIs, it is important to use an optimal volume of medication that will spread to the ventral epidural space at the lesion level.^[^1 3^]^ However, some study did not analyze spreading to ventral epidural space.^[^14 16^]^ Lastly, other study did not consider clinical variables. Although, contrast dye spread pattern known to well correlated with spread of medication,^[12]^ various factor such as viscosity, preinjected contrast volumes might be influence to spread of medication.^[^12 14 31^]^ Therefore, analyzing of contrast spread pattern with clinical variable is helpful to determine optimal volumes for CIEIs.^[^2 12^]^

The present study addressed all of these gaps. We performed CIEIs only at the C7 to T1 levels and using three different volumes of contrast dye in cervical degenerative disease. We also analyzed the detailed spread patterns of contrast using AP and CLO fluoroscopic images and fluoroscopic 3-dimensional reconstructed images. Additionally, we analyzed NRS score baseline, 1 and 3 months after CIEIs.

There were several limitations to our study of note. It was a retrospective review and did not employ controls, blinding, or randomization. Moreover, we performed CIEIs after cervical epidurograms. There is the possibility that the injected contrast dye may have affected the spread of medication and thus the NRS score.^[^14 21^]^ Another limitation is that we did not evaluate other clinical outcomes, such as neck disability index or patient global impression of change. Finally, the viscosity of the diluted contrast dye was different from medications injected in clinical practice. The viscosity of Omnipaque 300 at 37.0°C is 6.3 cP, whereas the viscosity of normal saline at 37.0°C is 0.8 cP.^[32]^

However, we primarily investigated the spread pattern of contrast dye by volume at the target level; these patterns may be helpful for the prediction of the spread of medication injected into cervical epidural space and the outcome of CIEIs in clinical practice. In addition, we assessed the NRS score after CIEIs at baseline and at 1 and 3 months follow-up, which may be helpful for the prediction of the spread of medication even if a cervical epidurogram is performed first. Lastly, we used diluted contrast than nondiluted contrast. Therefore, the possibility of contrast to affect the spread of medication was little than nondiluted contrast.

Despite these limitations, our results are clinically relevant. When performing CIEIs, 3 mL contrast dye is adequate for appropriate spread cephalad, bilaterally and throughout the ventral epidural space at the target level. Moreover, in clinical practice, this volume is sufficient for treating neck and upper-extremity pain induced by lower cervical degenerative disease.

## 5 Conclusions

There are no significant differences in contrast spread patterns such as cephalad ventral and bilaterally at target level among 3, 4.5, and 6 mL contrast medium in CIEIs. There are also no significant differences in NRS scores between these volumes. When performing CIEIs, 3 mL medication is sufficient volume for the treatment of neck and upper-extremity pain induced by lower cervical degenerative disease.
